# Understanding the role of parental decision-making and quality of clinical care in under-5 mortality in Western Kenya: an application of the Three Delays model

**DOI:** 10.1186/s12913-026-14634-8

**Published:** 2026-05-21

**Authors:** Kitiezo Aggrey Igunza, Beth A. Tippett Barr, Zachary J. Madewell, Dickens Onyango, Dickson Gethi, Joyce Were, Sarah Hawi Ngere, Harun Owuor, Janet Agaya, Peter Onyango Nyamthimba, Kephas Otieno, Broline S. Asuma, Sammy Khagayi, Florence Murila, Cynthia G. Whitney, Chris A. Rees, Dianna M. Blau, Richard Omore, Victor Akelo

**Affiliations:** 1 Kenya Medical Research Institute, P.O. Box 1578, Kisumu, 40100 Kenya; 2 Nyanja Health Research Institute, Salima, Malawi; 3 Centers for Disease Control and Prevention, Atlanta, GA USA; 4 Kisumu County Health Department, Kisumu, Kenya; 5 The University of Nairobi, Nairobi, Kenya; 6 Global Health Institute, Emory University, Atlanta, GA USA; 7 Division of Pediatric Emergency Medicine, Emory University School of Medicine, Atlanta, GA USA; 8 Children’s Healthcare of Atlanta, Atlanta, USA; 9 Liverpool School of Tropical Medicine, Liverpool, UK

**Keywords:** Three Delays model, Under-5 mortality, Kenya, Quality of care, Healthcare

## Abstract

**Background:**

Our objective was to assess the role of delayed healthcare seeking and clinical care among deceased children aged < 5 years enrolled in Child Health and Mortality Prevention Surveillance (CHAMPS) in Western Kenya.

**Methods:**

We conducted a retrospective descriptive analysis of verbal autopsies, health records, and postmortem-determined causes of death determined by an expert panel for decedent children aged < 5 years enrolled in CHAMPS-Kenya (2017–2022). We used the Three Delays framework to describe delays in (1) deciding to seek care, (2) reaching healthcare facilities, and (3) receiving adequate clinical care. Proportions were compared with Chi-square testing.

**Results:**

There were 814 enrolled cases in CHAMPS-Kenya and 611 had complete data and were included in our analysis. Nearly all cases (97.9%, *n* = 598/611) experienced at least one delay. Delayed caregivers’ decisions to seek clinical care increased by child’s age from 52.0% of < 1-day old deaths (*n* = 53/102) to 70.9% of child deaths among those aged 12–59 months (*n* = 83/117, *p* < 0.001), and were more common in malaria (52/63,82.5%) and malnutrition (51/64,79.7%) deaths than in deaths from other causes. Delays in receiving adequate clinical care were more common (84.9%, *n* = 519/560) than parental delays in deciding to seek care (58.9%, *n* = 366). All HIV deaths (31/31, 100.0%) and most malaria deaths (*n* = 57/63, 90.5%) experienced suboptimal or delayed clinical care.

**Conclusions:**

Nearly all young children who died experienced delays in seeking and receiving adequate clinical care prior to their death. Reducing childhood mortality requires a multi-pronged approach including caregiver education on the detection and response to danger signs, availing a functional referral system, and providing adequate clinical care.

## Background

Despite dramatic reductions in global annual under-5 mortality from 9.21 million (75 deaths/1000 live births) in 2000 to 4.66 million (35.7deaths/1000 live births) in 2021, additional efforts informed by current data are needed at global, regional, and country levels to end preventable deaths among young children, especially in areas with the highest burden [1, 2]. Estimates of under-5 mortality in 2021 are only 5 deaths/1,000 live births in high-income countries yet there are 74 deaths/1,000 live births in sub-Saharan Africa (SSA) [3]. These rates demonstrate that augmented efforts are needed to attain the United Nations’ Sustainable Development Goal (SDG) to end all preventable deaths among children aged < 5 years by 2030 [3].

Kenya’s overall mortality rate among children aged < 5 years is 41/1000 live births, but variation at subnational level indicates that the Nyanza region in Western Kenya has much higher mortality rates than the national average at 72 deaths/1,000 live births [4]. Kenya’s stillbirth rate is also high at 38 stillbirths per 1000 deliveries [5].

Persistently high childhood mortality rates in Western Kenya can be largely attributed to socioeconomic and geographic inequities that impede accessibility to, and utilization of, maternal, newborn, and child health services [6]. Previous efforts to prevent childhood mortality have included promoting healthcare service access (e.g., enhanced community case management of common illnesses) [7] and addressing shortfalls in the quality of service provided through community and structural interventions (e.g., promotion of adherence to clinical care guidelines and structured protocols) [8]. However, these interventions have unfortunately failed to achieve the expected reduction in mortality among children aged < 5 years [9]. A deeper understanding of the specific gaps in the seeking and provision of health care is needed to inform interventions that can prevent deaths among children aged < 5 years.

The Three Delays model is a theoretical framework that has been used to study timeliness of access to health care and inform health policy, [10]. and to global health emergencies [11]. This model includes three stages: (1) the delay in deciding to seek formal clinical care, (2) the delay in identifying and reaching a health facility, and (3) the delay in receiving timely and adequate clinical care upon arrival at health facilities [10, 12, 13]. 

Here, we applied the Three Delays model in a high-mortality setting in Western Kenya, with the goal of improving our understanding of gaps in the continuum of care from home to facility and identify the specific points where delays in receiving clinical care occur among children aged < 5 years who died in Western Kenya.

## Methods

### Study setting and design

This is a retrospective descriptive study using data collected in the Child Health and Mortality Prevention Surveillance (CHAMPS-Kenya) study. CHAMPS-Kenya is an ongoing program, established in 2017 in Manyatta within Kisumu County and Karemo within Siaya County in Western Kenya to collect comprehensive and high-quality data on causes of stillbirths and deaths among children aged < 5 years, in the community and health facilities. The total population under CHAMPS surveillance in Manyatta is 75,627 and that of Karemo is 100,703 [14]. The program is implemented by the Kenya Medical Research Institute (KEMRI) in collaboration with the Ministry of Health (MOH) and receives technical support from Emory University, Liverpool School of Tropical Medicine, and the US Centers for Disease Control and Prevention (CDC). We assessed deaths occurring between 17th May 2017 and 30th June 2022.

### Study procedures

CHAMPS conducts community and hospital surveillance and enrolls stillbirths and deaths in children aged < 5 years to determine their definitive causes of deaths through extensive postmortem testing. A detailed description of CHAMPS procedures has been documented previously [15–19]. Briefly, caregivers of the decedent children provided written informed consent prior to participation in this study. Although this was a retrospective study, data on delays were collected from several sources in order to identify all possible delays that may have occurred prior to the child’s death. These include detailed data from verbal autopsies, clinical records, and postmortem test results for each enrolled case which undergo rigorous case-by-case review by a local expert panel (i.e., the Determination of Cause of Death [DeCoDe] panel). The DeCoDe panel determined the immediate (the disease or complication which directly preceded or directly led to death), morbid/intervening, and underlying causes of death (disease or injury that initiated the chain of events leading directly to death, or circumstances of accident or violence which produced the fatal injury) [17]. The panel also identified other modifiable or preventable barriers to accessing timely quality care and provided public health recommendations on how deaths could be prevented [20]. The panel defined suboptimal/adequate clinical care as lack of adherence to Kenya national guidelines, inadequate documentation, or delays in healthcare administration. A data collection form was iteratively developed to prospectively quantify the frequency of each specific delay that was identified in DeCoDe meetings.

### Theoretical framework

**Fig. 1:**
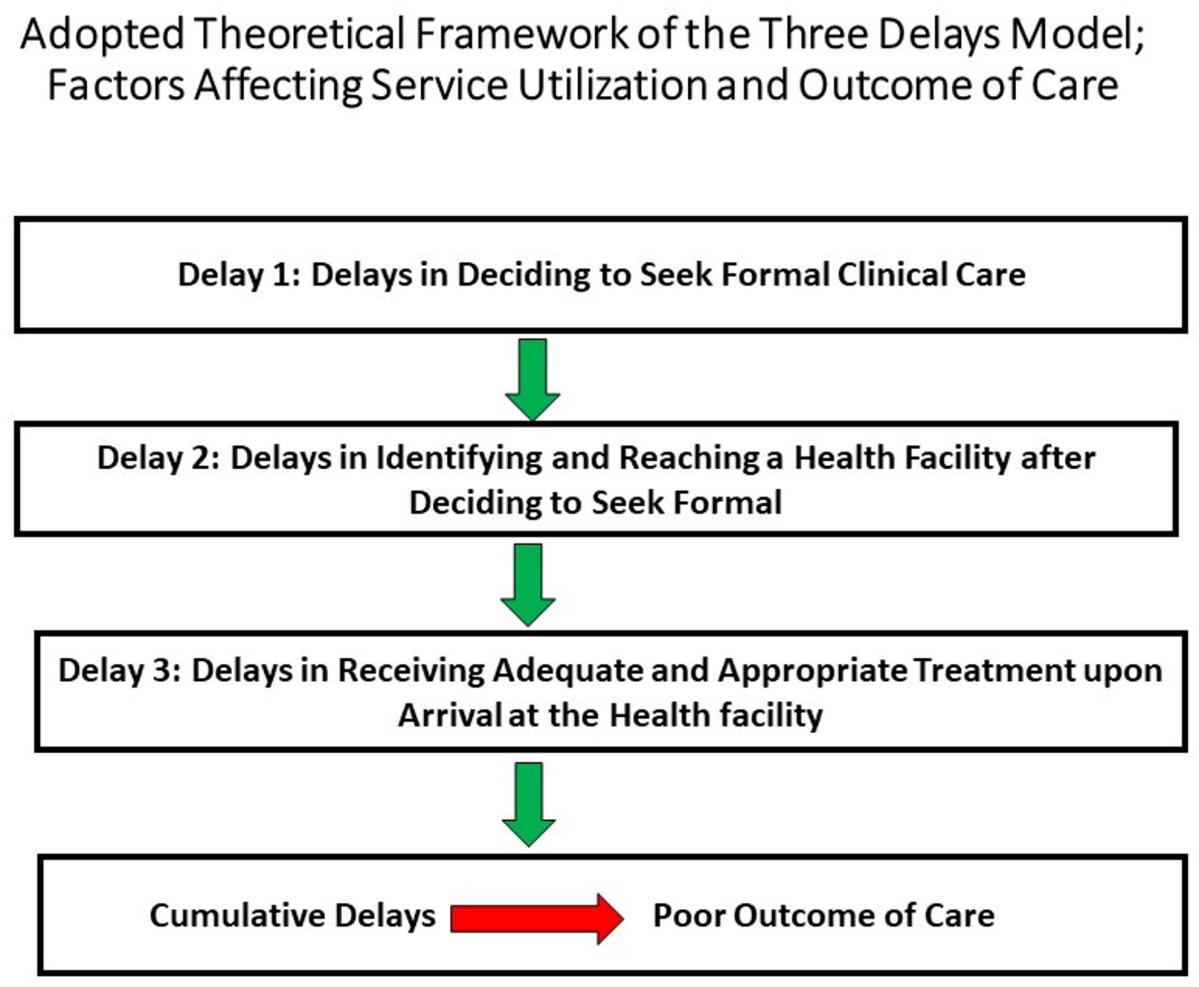
An adopted theoretical framework of the Three Delays model [12]

Our study adopted the Three Delays model (Fig. 1) to explore factors that contributed to delays along the health seeking pathway. These are delays in (1) deciding to seek appropriate medical care, (2) reaching an appropriate health facility, and (3) receiving adequate clinical care when a facility is reached. In past studies, the Three Delays model has been used to understand delays that contribute to maternal mortality [21], adverse events in surgical care [22], adult mortality in resource-limited settings [23, 24], and among children who required urgent medical attention [25–27]. Our study applied the Three Delays model to stillbirths and deaths among children aged < 5 years to identify the three critical phases that have direct consequences on child survival.

For the purposes of this study, we identified delays for each death using the Three Delays model based on the reviewed verbal autopsy, clinical, postmortem test results, and DeCoDe panel discussions. For stillbirths and neonatal deaths, maternal records were also examined for evidence of delays. Stillbirths and deaths among children aged < 5 years were included in our analyses if a cause of death was determined but excluded if a cause of death was indeterminate.

### Data management and analysis

All data were kept in a password-protected database in Kobo Toolbox. All data on delays was collected as free text in the site’s main data collection tool and was reviewed to quantify reported delays. A descriptive analysis of the data to determine the cumulative frequencies and proportions of each delay overall, by age groups, and by cause of death done per the Three Delays model. Average delays experienced by each case per cause of death were calculated. Subgroup comparisons of delays by age groups and causes of death were made using Chi-square testing. All statistical analyses were performed using R, version 4.1.2 (R Foundation for Statistical Computing).

## Results

During the study period, 814 cases were enrolled in CHAMPS-Kenya. Of these, 183 had incomplete data that precluded the identification of delays, 20 cases had an undetermined cause of death, and 611 deaths were included in our analysis. Of these, 27.8% (*n* = 170) were stillbirths, 30.4% (*n* = 186) were deaths among neonates aged 0–28 days, and 41.7% (*n* = 255) were deaths of infants and children aged 1–59 months (Table 1). Nearly two thirds of neonatal deaths (*n* = 170/272, 62.5%) occurred on the first day of life, and approximately one-quarter (23.7%, *n* = 145) occurred at home. Around half of the deaths were male (53.4%, *n* = 326), and 54.2% (*n* = 331) lived in urban areas.

**Table 1:** Proportions of the 3 delays against age at death, sex, residence and location of death

	Deaths* N (%)	Deaths with no delay** *n* (%)	Deaths with any delay *N* (%)	1st delay *N* (%)	2nd delay *n* (%)	3rd delay *N* (%)	Total number of delays	Ratio of delays to deaths
*Overall*	611 (100.0)	13 (2.1)	*598 (97.9)*	*366 (59.9)*	*43 (7.0)*	*519 (84.9)*	928	1.5
*Age group*			*p* = 0.87	*p* = 0.001	*p* = 0.01	*p* = 0.96		
Stillbirth	170 (27.8)	3 (1.8)	167 (98.2)	93 (54.7)	6 (3.5)	142 (83.5)	241	1.4
< 1 day	102 (16.7)	3 (2.9)	99 (97.1)	53 (52.0)	7 (6.9)	88 (86.3)	148	1.5
Neonate (1-28days)	84 (13.7)	1 (1.2)	83 (98.8)	42 (50.0)	2 (2.4)	71 (84.5)	115	1.4
Infant (1–11 mos)	138 (22.6)	4 (2.9)	134 (97.1)	95 (68.8)	13 (9.4)	117 (84.8)	225	1.6
Child (12–59 mos)	117 (19.1)	2 (1.7)	115 (98.3)	83 (70.9)	15 (12.8)	101 (86.3)	199	1.7
*Sex*			*p* = 0.98	*p* = 0.877	*p* = 0.643	*p* = 0.678		
Male	326 (53.4)	7 (2.1)	319 (97.9)	196 (60.1)	21 (6.4)	275 (84.4)	492	1.5
Female	284 (46.5)	6 (2.1)	278 (97.9)	169 (59.5)	21 (7.4)	243 (85.6)	433	1.5
Undetermined	1 (0.2)	0 (0.0)	1 (100.0)	1 (100.0)	1 (100.0)	1 (100.0)	3	3.0
*Residence*			*p* = 0.981	*p* = 0.02	*p* = 0.05	*p* = 0.19		
Urban	331(54.2)	7 (2.1)	324 (97.9)	184 (55.6)	17 (5.1)	287 (86.7)	488	1.5
Rural	280 (45.8)	6 (2.1)	274 (97.9)	182 (65.0)	26 (9.3)	232 (82.9)	440	1.6
*Location at Death*			*p* = 0.96	*p* < 0.001	*p* < 0.001	*p* < 0.001		
Facility	466 (76.3)	10 (2.1)	456 (97.9)	247 (53.0)	21 (4.5)	409 (87.8)	677	1.5
Community	145 (23.7)	3 (2.1)	142 (97.9)	3 (2.1)	119 (82.1)	22 (15.2)	110	0.8

*Deaths are reported as column percentages, while all others are row percentages

**As deaths can experience multiple delays, each delay category was compared individually to ‘no delay’, providing p-values from chi-square testing for each

### Frequency of delays

Nearly all (97.9%, *n* = 598/611) deaths experienced at least one delay, with an average ratio of 1.5 delays per death. There was a higher ratio of delays per deaths in children aged 12–59 months than the other age groups (Table 1). Over half of the deaths (59.9%, *n* = 366) experienced delays in caregivers deciding to seek clinical care, 7.0% (*n* = 43) experienced delays in getting to a health facility, and 84.9% (*n* = 519) experienceddelays inin receiving adequate clinical care once at a health facility. Delays in caregivers deciding to seek clinical care and delays in getting to a health facility were more common in rural versus urban areas (*p* = 0.02 and *p* = 0.05, respectively), but delays in receiving adequate clinical care was not different by location (*p* = 0.19; Table 1).

Of those who died in a health facility, over half experienced delays in caregivers deciding to seek clinical care (*n* = 247/466, 53.0%) compared to 2.1% (3/145) of those who died in the community (*p* < 0.001; Table 1). Of those who died in the community, over 80% experienced delay in getting to a health facility (*n* = 119/145), compared to 4.5% of those who died in a facility (*n* = 21/466, *p* < 0.001). Nearly 90% of those dying in a health facility experienced suboptimal or delayed clinical care delays in receiving adequate clinical care once at a health facility (*n* = 409/466, 87.8.%).

### Reasons for, and interactions between, the Three Delays

The most commonly identified reason for delaying the decision to seek clinical care was caregivers’ inability to recognize danger signs (*n* = 77/318, 24.2%) followed by the decision to initially use traditional medicine (*n* = 66/318, 20.8%) before seeking formal healthcare (Table 2). Failure to recognize danger signs was common among stillbirths (59.0%, *n* = 46/78), and seeking traditional medicine as a first therapeutic option was common among children aged 12–59 months (39.0%, *n* = 30/77) whose caregivers delayed in deciding to seek clinical care. Delays reaching a health facility were primarily attributed to safety concerns related to night travel (69.8%, *n* = 30/43) or difficulties in securing transport, often at night (23.3%, *n* = 10/43).

**Table 2:** Type of delay by age group for all deaths

		Stillbirth *n* = 172	< 1 day *n* = 101	Neonate *n* = 84	Infant *n* = 138	Child *n* = 117	Total Delays* *n* = 880**
		*n*(%)	*n* (%)	*n* (%)	*n* (%)	*n* (%)	*n* (%)
No delay		3 (1.8)	3 (3.0)	1 (1.2)	4 (2.9)	2 (1.7)	na
*1st Delay: Delayed decision to seek clinical care*		*78 (45.3)*	*44 (43.6)*	*33 (39.3)*	*86 (62.3)*	*77 (65.8)*	*318 (36.1)*
Didn’t recognize danger signs		46 (59.0)	8 (18.2)	10 (30.3)	6 (7.0)	7 (9.1)	77 (24.2)
Underestimated risk of illness		18 (23.1)	11 (25.0)	3 (9.1)	11 (12.8)	11 (14.3)	54 (17.0)
Used traditional medicine first		2 (2.6)	1 (2.3)	7 (21.2)	26 (30.2)	30 (39.0)	66 (20.8)
Didn’t know malnutrition home-care skills		0 (0.0)	0 (0.0)	1 (3.0)	19 (22.1)	15 (19.5)	35 (11.0)
Didn’t understand risk of incomplete ANC visits		8 (10.3)	13 (29.5)	4 (12.1)	5 (5.8)	2 (2.6)	32 (10.1)
Decided to deliver at home		4 (5.1)	11 (25.0)	5 (15.2)	6 (7.0)	1 (1.3)	27 (8.5)
Didn’t know post-discharge home care skills		0 (0.0)	0 (0.0)	2 (6.1)	8 (9.3)	6 (7.8)	16 (5.0)
Didn’t understand risk of incomplete vaccination		0 (0.0)	0 (0.0)	1 (3.0)	5 (5.8)	5 (6.5)	11 (3.5)
*2nd Delay: Delay in seeking care after deciding to do so*		*6 (3.5)*	*7 (6.9)*	*2 (2.4)*	*13 (9.4)*	*15 (12.8)*	*43 (4.9)*
Lack of personal safety for night travel		5 (83.3)	7 (100.0)	1 (50.0)	8 (61.5)	9 (60.0)	30 (69.8)
Couldn’t get transport quickly		1 (16.7)	0 (0.0)	1 (50.0)	3 (23.1)	5 (33.3)	10 (23.3)
Competing obligations (i.e., Child care)		0 (0.0)	0 (0.0)	0 (0.0)	2 (15.4)	1 (6.7)	3 (7.0)
*3rd Delay: Delay in receiving optimal clinical care*		*142 (83.5)*	*88 (87.1)*	*71 (84.5)*	*117 (84.8)*	*101 (86.3)*	*519 (59.0)*
Inadequate documentation to monitor care		77 (54.2)	42 (47.7)	26 (36.6)	14 (12.0)	12 (11.9)	171 (32.9)
Clinical guidelines not followed		25 (17.6)	8 (9.1)	21 (29.6)	65 (55.6)	39 (38.6)	158 (30.4)
Referral late or incomplete		25 (17.6)	24 (27.3)	17 (23.9)	17 (14.5)	29 (28.7)	112 (21.6)
Commodities out of stock		6 (4.2)	2 (2.3)	4 (5.6)	10 (8.5)	12 (11.9)	34 (6.6)
Lack of a contingency fund		5 (3.5)	4 (4.5)	1 (1.4)	7 (6.0)	5 (5.0)	22 (4.2)
Lab/Radiology support not available/ not used		4 (2.8)	3 (3.4)	2 (2.8)	4 (3.4)	4 (4.0)	17 (3.3)
Providers not respectful of patient/parent		0 (0.0)	5 (5.7)	0 (0.0)	0 (0.0)	0 (0.0)	5 (1.0)

*Each death could experience multiple delays (i.e., Total delays > total deaths)

**48 1st delays did not have a specific reason provided, so the total delays here are 880 compared to the 928 delays recorded in Table 1

Delays in receiving adequate clinical care once at a facility comprised 59.0% (519/880) of all delays, and were most commonly linked to inadequate documentation to monitor care (32.9%, *n* = 171/519), non-compliance with national pediatric clinical care guidelines (30.4%, *n* = 158/519), and late or incomplete referrals to a higher level of care (21.6%, *n* = 112/519) (Table 2). Poor documentation of clinical care was determined in over half of the stillbirth cases (54.2%, *n* = 77/142) and 11.9% (*n* = 12/101) of children aged 12–59 months (Table 2). Failure to adhere to national pediatric clinical guidelines occurred in over half the infant deaths aged 1–11 months (55.6%, *n* = 65/117) and over a third of child deaths aged 12–59 months (38.6%, *n* = 39/101).

**Fig. 2:**
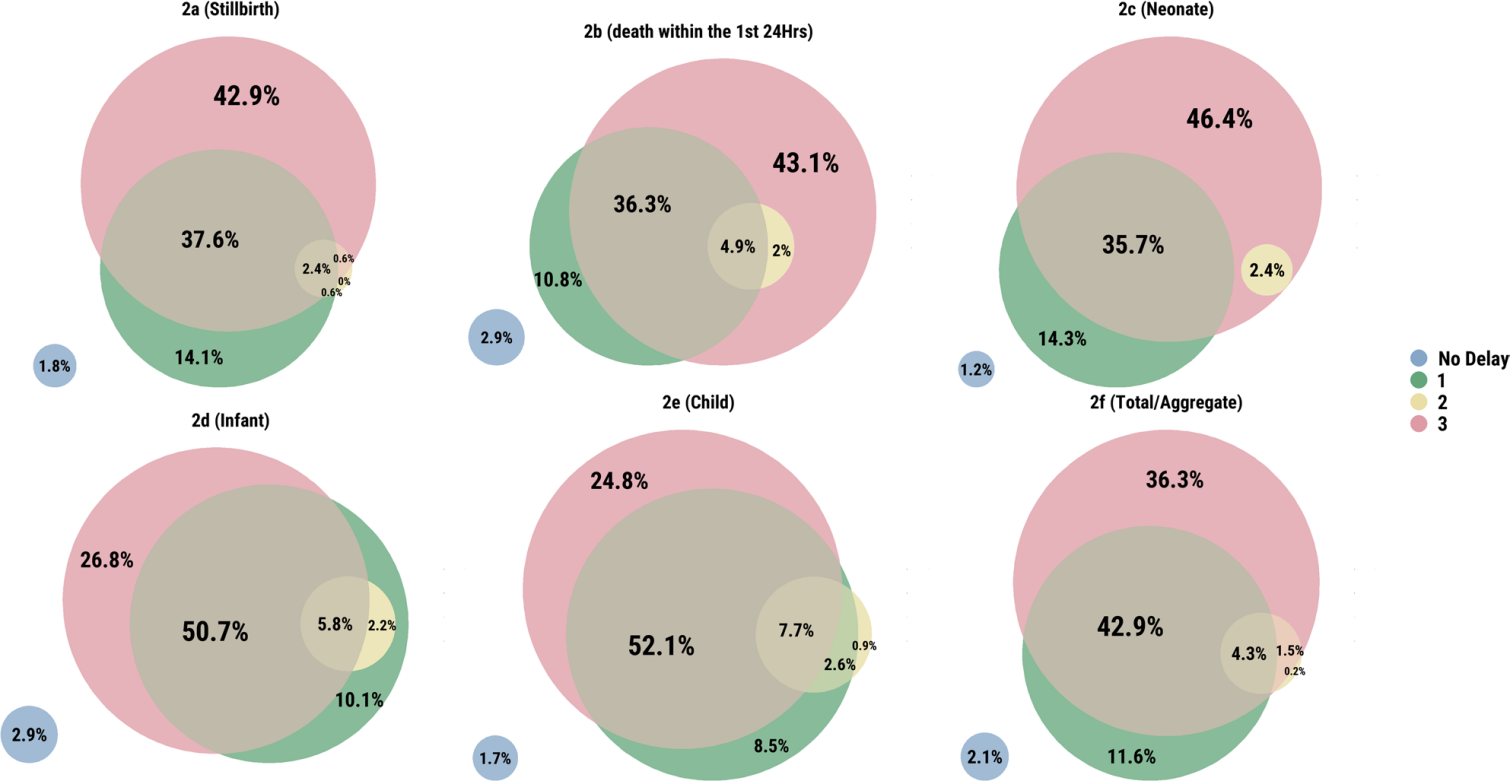
Proportional contribution and interaction between each of the Three Delays contributing to deaths among children aged < 5 years. *Size of the circles is directly proportional to the frequency of delays experienced at a given age group

The interaction of the three delays was similar across age groups (Fig. 2), although suboptimal clinical care was more commonly the single delay in neonatal cases than other age groups (46.4%), and least common among infants and children (26.8% and 24.8%, respectively). Delays in getting to a health facility were most commonly observed in child deaths (i.e., those aged 12–59 months), but in all age groups commonly occurred with one or both of the other delays. Across all age groups, less than 3% of deaths experienced no delay. For all deaths that occurred beyond 24 h of birth through child deaths, the frequency of caregiver delays in seeking formal clinical care was more common among children than neonates.

### Variation in delays by causes of death within age groups

The most common immediate or underlying causes of death varied by age group (Table 3). The average ratio of delays per cause of death varied the most among infants (0.6 for pneumonia and 2.1 for sepsis). Infants had the lowest average ratio of delays to deaths.

**Table 3:** Burden and distribution of the Three Delays in the top five immediate and underlying causes of death for each age group

		Total top 5 immediate and/or underlying causes of death (*n* = 616)*	Delay 1** (*n* = 367)	Delay 2 (*n* = 46)	Delay 3 (*n* = 510)	Total delays per Cause of Death	Ratio of Delays to Causes of Death
*Stillbirths*		*170 (27.6)*	*95 (55.9)*	*6 (3.5)*	*145 (84.7)*	*245*	*1.4*
Intrauterine hypoxia / Asphyxia		162 (95.3)	92 (56.8)	6 (3.7)	137 (84.6)	235	1.5
Intrauterine Infection		4 (2.4)	2 (50.0)	0 (0.0)	3 (75.0)	5	1.3
Low birthweight		2 (1.2)	0 (0.0)	0 (0.0)	2 (100.0)	2	1.0
Cytomegalovirus		1 (0.6)	1 (100.0)	0 (0.0)	1 (100.0)	2	2.0
Meconium Aspiration Syndrome		1 (0.6)	0 (0.0)	0 (0.0)	1 (100.0)	1	1.0
*Death in 1st 24 h*		*91 (14.8)*	*47 (51.6)*	*6 (6.6)*	*83 (91.2)*	*136*	*1.5*
Intrauterine hypoxia / Asphyxia		49 (53.8)	21 (42.9)	1 (2.0)	42 (85.7)	64	1.3
Respiratory distress syndrome		23 (25.3)	18 (78.3)	3 (13.0)	23 (100.0)	44	1.9
Meconium aspiration syndrome		9 (9.9)	2 (22.2)	1 (11.1)	9 (100.0)	12	1.3
Sepsis		5 (5.5)	2 (40.0)	1 (20.0)	5 (100.0)	8	1.6
Low birthweight		5 (5.5)	4 (80.0)	0 (0.0)	4 (80.0)	8	1.6
*Neonate*		*71 (11.5)*	*40 (56.3)*	*2 (2.8)*	*69 (97.2)*	*111*	*1.7*
Sepsis		30 (42.3)	24 (80.0)	0 (0.0)	30 (100.0)	63	2.1
Pneumonia		14 (19.7)	7 (50.0)	1 (7.1)	14 (100.0)	22	1.6
Intrauterine hypoxia / Asphyxia		11 (15.5)	1 (9.1)	0 (0.0)	11 (100.0)	13	1.2
Respiratory distress syndrome		9 (12.7)	4 (44.4)	1 (11.1)	7 (77.8)	12	1.3
Low birthweight		7 (9.9)	4 (57.1)	0 (0.0)	7 (100.0)	11	1.6
*Infant*		*128 (20.8)*	*69 (53.9)*	*11 (8.6)*	*81 (63.3)*	*161*	*1.3*
Pneumonia		43 (33.6)	12 (27.9)	1 (2.3)	13 (30.2)	26	0.6
Malnutrition		39 (30.5)	31 (79.5)	6 (15.4)	36 (92.3)	73	1.9
Gastroenteritis		18 (14.1)	7 (38.9)	1 (5.6)	10 (55.6)	18	1.0
Malaria		17 (13.3)	14 (82.4)	3 (17.6)	12 (70.6)	29	1.7
HIV Infection		11 (8.6)	5 (45.5)	0 (0.0)	10 (90.9)	15	1.4
*Child*		*156 (25.3)*	*116 (74.4)*	*21 (13.5)*	*132 (84.6)*	*269*	*1.7*
Malaria		46 (29.5)	36 (78.3)	8 (17.4)	35 (76.1)	79	1.7
Pneumonia		34 (21.8)	21 (61.8)	5 (14.7)	28 (82.4)	54	1.6
Sepsis		31 (19.9)	22 (71.0)	2 (6.5)	30 (96.8)	54	1.7
Malnutrition		25 (16.6)	24 (96.0)	5 (23.8)	20 (80.0)	49	2.0
HIV infection		20 (12.8)	13 (65.0)	1 (5.0)	19 (95.0)	33	1.7

*Deaths are reported in column percentages to allow comparison of disease burden by age and cause of death. Number of causes of death exceed total deaths analyzed as one death may have both immediate and underlying causes included in this analysis

**Delays are reported by row percentages to allow relative comparison of delays by age and cause of death. Number of delays may be double-counted when applying to a death with multiple causes (i.e., Delays apply to the cause of death, not to the death)

Over three-quarters of stillbirths resulting from intrauterine hypoxia experienced suboptimal clinical care (*n* = 137/162, 84.6%), and all neonatal deaths from respiratory distress syndrome, meconium aspiration syndrome, pneumonia, and sepsis received sub-optimal clinical care. In neonatal sepsis deaths, 80% (*n* = 24/30) also experienced a delay in parental decision-making to seek clinical care. All neonates who had low birthweight received suboptimal clinical care.

Infant deaths from malaria (14/17, 82.4%) and malnutrition (31/39, 79.5%) experienced the greatest delays in parental decision-making, and the greatest burden of suboptimal clinical care (70.6% and 92.3%, respectively). The same pattern of parental delay was observed in child deaths from malaria and malnutrition (78.3% and 96.0% respectively).

### Reasons for delays by cause of death

There was variation in the frequency of delayed parental decision-making to seek care by cause of death (ranging from 44.6% [*n* = 95/213] of birth asphyxia deaths to 82.5% [*n* = 52/63] of malaria deaths, Table 4). Delays in caregivers deciding to seek clinical care occurred in 79.7% of malnutrition deaths, with 40% of mothers reportedly lacked the necessary malnutrition home-care skills. For both malnutrition and malaria, approximately one third of mothers chose to use traditional medicine before seeking clinical care.

**Table 4:** Type of delay by cause of death for the top five causes of death across all age groups

Delay	Deaths (*n* = 560)*	Hypoxia/ Asphyxia (*n* = 213)	Pneumonia (*n* = 91)	Sepsis (*n* = 118)	Malnutrition (*n* = 64)	Malaria (*n* = 63)	Respiratory distress syndrome (*n* = 39)	HIV (*n* = 31)	Low birthweight (*n* = 24)	Total delays**
	*n*	*n*	*n*	*n*	*n*	*n*	*n*	*n*	*n*	*n*
*1st Delay: Delayed decision to seek clinical care*	*318 (52.0)*	*95 (44.6)*	*57 (62.6)*	*62 (52.5)*	*51 (79.7)*	*52 (82.5)*	*23 (59.0)*	*16 (51.6)*	*14 (58.3)*	*317 (51.9)*
Didn’t recognize danger signs	77 (24.2)	47 (49.5)	6 (10.5)	11 (17.7)	1 (2.0)	4 (7.7)	2 (8.7)	1 (6.3)	4 (28.6)	69 (21.8)
Underestimated risk of illness	54 (17.0)	25 (26.3)	7 (12.3)	6 (9.7)	2 (3.9)	9 (17.3)	4 (17.4)	1 (6.3)	1 (7.1)	49 (15.5)
Used traditional medicine first	66 (20.8)	4 (4.2)	21 (36.8)	17 (27.4)	17 (33.3)	16 (30.8)	1 (4.3)	3 (18.8)	2 (14.3)	75 (23.7)
Didn’t know malnutrition home-care skills	35 (11.0)	0 (0.0)	10 (17.5)	12 (19.4)	20 (39.2)	9 (17.3)	0 (0.0)	3 (18.8)	0 (0.0)	51 (16.1)
Didn’t understand risk of incomplete ANC visits	32 (10.1)	10 (10.5)	4 (7.0)	4 (6.5)	1 (2.0)	2 (3.8)	8 (34.8)	3 (18.8)	3 (21.4)	21 (6.6)
Didn’t understand risk of incomplete vaccination	11 (3.5)	0 (0.0)	2 (3.5)	5 (8.1)	2 (3.9)	5 (9.6)	0 (0.0)	4 (25.0)	1 (7.4)	14 (4.4)
Decided to deliver at home	27 (8.5)	9 (9.5)	2 (3.5)	4 (6.5)	2 (3.9)	2 (3.8)	7 (30.4)	0 (0.0)	2 (14.3)	19 (6.0)
Didn’t know post-discharge home care skills	16 (5.0)	0 (0.0)	5 (8.8)	3 (4.8)	6 (11.8)	5 (9.6)	1 (4.3)	1 (6.3)	1 (7.1)	19 (6.0)
*2nd Delay: Delay in reaching care*	*43 (7.0)*	*8 (3.8)*	*7 (7.7)*	*5 (4.2)*	*11 (17.2)*	*11 (17.5)*	*4 (10.3)*	*1 (3.2)*	*1 (4.2)*	*42 (6.9)*
Lack of personal safety for night travel	10 (23.3)	6 (75.0)	4 (57.1)	2 (40.0)	6 (54.5)	7 (63.6)	4 (100.0)	1 (100.0)	1 (100.0)	25 (59.5)
Couldn’t get transport quickly	30 (69.8)	2 (25.0)	3 (42.9)	2 (40.0)	4 (36.4)	3 (27.3)	0 (0.0)	0 (0.0)	0 (0.0)	14 (33.3)
Competing obligations (i.e., Childcare)	3 (7.0)	0 (0.0)	0 (0.0)	1 (20.0)	1 (9.1)	1 (9.1)	0 (0.0)	0 (0.0)	0 (0.0)	3 (7.1)
*3rd Delay: Delay in receiving optimal clinical care*	*517 (84.6)*	*195 (91.5)*	*79 (86.8)*	*102 (86.4)*	*56 (87.5)*	*57 (90.5)*	*33 (84.6)*	*31 (100.0)*	*22 (91.7)*	*489 (80.0)*
Clinical guidelines not followed	158 (30.6)	27 (13.8)	38 (48.1)	47 (46.1)	33 (58.9)	24 (42.1)	7 (21.2)	20 (64.5)	7 (31.8)	169 (34.6)
Inadequate documentation to monitor care	171 (33.1)	108 (55.4)	13 (16.5)	21 (20.6)	4 (7.1)	5 (8.8)	10 (30.3)	3 (9.7)	7 (31.8)	151 (30.9)
Referral late or incomplete	112 (21.7)	36 (18.5)	14 (17.7)	20 (19.6)	11 (19.6)	19 (33.3)	12 (36.4)	2 (6.5)	5 (22.7)	100 (20.4)
Commodities out of stock	32 (6.2)	8 (4.1)	4 (5.1)	8 (7.8)	3 (5.4)	7 (12.3)	1 (3.0)	5 (16.1)	2 (9.1)	30 (6.1)
Lack of a contingency fund	22 (4.3)	10 (5.1)	5 (6.3)	2 (2.0)	3 (5.4)	1 (1.8)	0 (0.0)	1 (3.2)	0 (0.0)	21 (4.3)
Lab/Radiology support not available or used	17 (3.3)	4 (2.1)	5 (6.3)	4 (3.9)	2 (3.6)	1 (1.8)	1 (3.0)	0 (0.0)	1 (4.5)	16 (3.3)
Providers not respectful of patient/parent	5 (1.0)	2 (1.0)	0 (0.0)	0 (0.0)	0 (0.0)	0 (0.0	2 (6.1)	0 (0.0)	0 (0.0)	2 (0.4)

*Column percentages shown by delay

Greater delays in getting to a health a facility were also observed for malnutrition (17.2%) and malaria (17.5%), with half to two thirds of caregivers citing unsafe or delayed access to transport at night being the main reasons for delays in getting to healthcare facilities.

Suboptimal clinical care was documented in 84.6% of deaths (*n* = 517/560), including 100% of HIV-related deaths, and nearly all hypoxia/asphyxia (91.5%), malaria (90.5%), and low birthweight (91.7%) deaths. The most common contributors to delays in receiving adequate clinical care once in a health facility were failure to follow clinical guidelines (34.6%) and inadequate documentation for monitoring ongoing clinical care (30.9%). Failure to follow clinical guidleines varied widely by cause of death, from 13.8% of hypoxia deaths to 64.5% of HIV deaths.

## Discussion

In our study that aimed to assess the role of delayed healthcare seeking and clinical care among 611 deceased children aged < 5 years in Western Kenya, nearly all stillbirths and childhood deaths occurred following at least one delay in seeking or receiving optimal clinical care. Delays in receiving optimal clinical care once at a facility were common. Older children were most likely to experience delayed parental decision-making, as were malnutrition and malaria deaths.

Our results suggest that the majority of childhood deaths in Western Kenya occurred after delays in caregivers deciding to seek clinical care and following delays in receiving adequate clinical care once at a health facility. Perhaps health education on the recognition and response to danger signs in childhood illness should be enhanced with monitoring and evaluation of its impact in Western Kenya. The most common reason for a delay in deciding to seek clinical care included not recognizing danger signs in late pregnancy. Health programs and healthcare providers can improve maternal knowledge on birth preparedness through strengthening proven, and accessible, health education strategies [28]. The delay in deciding to seek clinical care was also most common in malaria and malnutrition deaths in both infants and children, indicating that caregivers commonly underestimate the risk of common acute disease and chronic conditions. Prior studies have provided clear examples of interventions, which successfully address most of the delays we identified, but these interventions either have not been brought to scale in our resource-constrained setting or are not implemented with adequate oversight to ensure optimal impact.

In this study, traditional medicine was a first choice for 20% of all children who died, and was more common among children than among infants or neonates. Prior qualitative study findings in Western Kenya suggest that traditional medicine may be used due to caregiver preference based on their beliefs on disease etiology [29], and other studies have demonstrated that the use of home initiated interventions with herbs or medicines may be common, leading to further delays in definitive care [30]. Lower costs of traditional medicine compared to the formal health system may also be a reason for its frequent use as an initial therapy [31, 32]. 

For caregivers who have transportation but still delay seeking care, health authorities may focus on incorporating effective referral systems embedded within community health systems. To address challenges in transport to health facilities, health authorities could invest resources in 24-hour toll free lines, functional ambulances, and corresponding trained personnel to help minimize preventable child deaths [33, 34]. 

The most common contribution to preventable deaths was delayed and suboptimal clinical care within health facilites across all ages and causes of death, which suggests that systemic issues in the health system contribute to child mortality more than delayed parental decision-making. National clinical guidelines exist to improve the quality of care and to promote patient safety by promoting evidence-based medicine [35]. Within health facilities, there was suboptimal clinical management manifesting as poor adherence to national clinical care guidelines. Our findings suggest that timely administration of medications and appropriate therapy are needed in order to reduce childhood mortality. These findings corroborate those of another study which revealed poor provider adherence to the World Health Organization treatment guidelines in treatment of leading causes of death [36].

Stillbirths and neonatal deaths may be prevented by improving clinical care in pregnancy and during delivery. The Kenya National Guidelines for quality obstetrics indicate that a properly filled partograph gives graphical guidance on fetal heart rate, amniotic fluid, fetal descent, head molding, rapture of membranes, maternal blood pressure, pulse rate, uterine contractions, and cervical dilatation, which are essential in informing timely decision making for intervention [37]. The findings of our study align with those of a study conducted in Ethiopia in which recommendations were made for health authorities to facilitate the availability of partographs and to promote its routine use and supervised utilization [38]. 

Another recent analysis conducted on CHAMPS data generated from seven-countries, including data from Western Kenya, determined that over three-quarters of stillbirths and deaths among children aged < 5 years were preventable through timely access to higher quality clinical care [20]. This suggests that mortality among children aged < 5 years rates can be reduced further if targeted interventions are employed to enhance healthcare seeking and provision. Prior studies have demonstrated promising outcomes following health education [39], community health services [40, 41], prompt and safe night transport to health facilities [42–44], enhanced clinical oversight [45–48], refresher trainings [49], and perinatal mortality reviews [50–52]. Given the frequency of delays observed in our study, such interventions are needed and have yet to be implemented fully in resource-constrained settings such as our study area in Western Kenya.

### Limitations

Our study has some limitations. Most importantly, we used data abstracted from verbal autopsies and clinical records to assess delays. In the absence of specific questions at the time of data collection, we may not have documented all delays faced in each case and therefore under-estimated the frequency of delays. Thus, our findings should be considered a minimum estimate of delays experienced in our site in Kenya. Additionally, the burden and distribution of delays in our population of deceased children aged < 5 years may not reflect that of severely ill children who survived. Further studies are warranted to compare the frequency of delays among those who survive and those who die, so that interventions and resources can be optimally allocated.

## Conclusions

Our findings underscore the significance of addressing delays in parental decision-making, reaching health facilities, and receiving timely and optimal clinical care to reduce child mortality. Efforts are urgently needed to improve the implementation of already-proven, cost-efficient and nationally-endorsed interventions to reduce childhood mortality through implementation of proven interventions.

## Data Availability

The data utilized in this study contain potentially identifiable information and, as a result, are only partially available through reasonable request to the corresponding author.

## Abbreviations

SDG: Sustainable development goals
CHAMPS: Child Health and Mortality Prevention Surveillance
KEMRI: Kenya Medical Research Institute
MOH: Ministry of Health
CDC: Center for Disease Control
DeCoDe: Determination of cause of death
